# Anti-PD-1 Immunotherapy Combined With Stereotactic Body Radiation Therapy and GM-CSF as Salvage Therapy in a PD-L1-Negative Patient With Refractory Metastatic Esophageal Squamous Cell Carcinoma: A Case Report and Literature Review

**DOI:** 10.3389/fonc.2020.01625

**Published:** 2020-09-02

**Authors:** Xiangrong Zhao, Yuehong Kong, Liyuan Zhang

**Affiliations:** ^1^Department of Radiotherapy & Oncology, The Second Affiliated Hospital of Soochow University, Suzhou, China; ^2^Institute of Radiotherapy & Oncology, Soochow University, Suzhou, China

**Keywords:** immunotherapy, radiotherapy, PD-L1, esophageal squamous cell carcinoma, GM-CSF

## Abstract

Esophageal squamous cell carcinoma (ESCC) is a malignancy with poor prognosis, which is often diagnosed at a late stage. Effective treatment options are limited when patients fail standard systemic therapy. The application of PD-1 inhibitors have led to a paradigm shift in the treatment of ESCC, but its efficacy as monotherapy is limited. Previous studies have shown that the antitumor effects may be reinforced when a PD-1 inhibitor is combined with radiotherapy or GM-CSF. This study aimed to report a case of a patient about advanced unresectable ESCC negative expression of PD-L1, who experienced tumor progression after chemoradiotherapy and targeted therapy.A significant systemic effect was seen after PD-1 inhibitor combined with GM-CSF and stereotactic body radiotherapy (SBRT) for metastatic lesions, however, severe pneumonia occurred after the triple-combination therapy. This study also reviewed several reports about the efficacy and safety of combination therapy.

## Introduction

Esophageal carcinoma is the sixth leading cause of cancer-related death in the world, with high malignancy and poor prognosis (1). More than half of patients with ESCC were initially diagnosed in an advanced or metastatic stage and treated with platinum-based chemotherapy regimens, commonly combined with fluoropyrimidine or taxane, as the main treatment. However, the long-term survival of these regimens remains poor, and the overall survival (OS) is as short as 7.7–15.5 months (2–4). The treatment options are more limited if ESCC progresses during or after standard first-line chemotherapy. Single-agent second-line chemotherapy, such as irinotecan, is recommended, resulting in poor OS of approximately 5 months. In addition, the incidence of adverse events caused by chemotherapy is high, seriously affecting the quality of life of patients. Targeted therapy, such as apatinib or anlotinib, was approved as backline treatment in China, but it had no obvious breakthrough in efficacy, with a median OS of only 6 months (5, 6). Hence, it was believed that the treatment of advanced esophageal cancer had entered the bottleneck.

In 2019, pembrolizumab was officially approved for second-line and above treatment of PD-L1 positive combined positive score(CPS) ≥ 10 patients with advanced ESCC, which led to longer OS compared with chemotherapy (9.3 and 6.7 months, respectively) with statistical significance (7). However, the objective response rate (ORR) was only 6.4% in PD-L1-negative patients (7). Previous studies showed that the antitumor effects might be reinforced when a PD-1 inhibitor was combined with radiotherapy or GM-CSF. Recently, a substantial amount of data has emerged showing that stereotactic ablative radiotherapy (SABR), also known as SBRT, can enhance the immune system to kill tumors and achieve better tumor control. This reaction can be strengthened by the use of a PD-1 inhibitor or GM-CSF (8, 9). In addition, PD-L1-negative patients can benefit more from SBRT combined with a PD-1 inhibitor compared with PD-L1-positive ones (10). GM-CSF can promote the proliferation, maturation and migration of dendritic cells. Dendritic cells are antigen-presenting cells and play important roles in the anti-tumor effect of T cells (11). GM-CSF has also shown encouraging results in combination with a PD-1 inhibitor or radiotherapy in cancer treatment (12–14). The combination of PD-1 inhibitors with GM-CSF or radiotherapy induced remarkable antitumor immune effects (14) and produced objective abscopal effects in some patients with metastatic solid tumors (15).

In the present case, the tumor burden was significantly reduced by the triple-combination treatment, the suggested mechanisms might involve radio-sensitization of immunotherapy.

## Case Presentation

**Figure 1:**
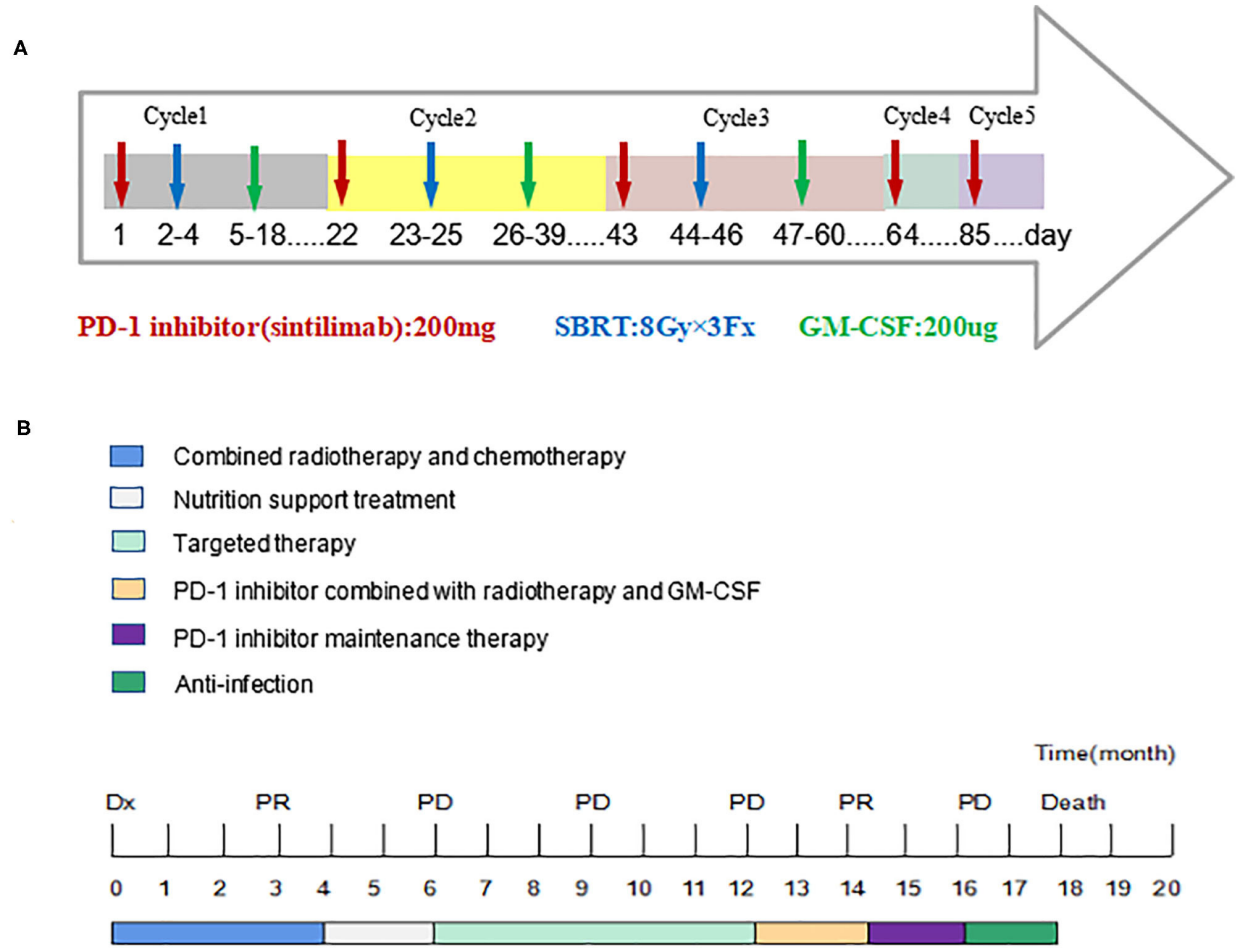
Timeline of five cycles treatment for the patient and the whole treatment process. Dx indicates diagnosis. PR, Partial Response; PD, Progressive Disease. **(A)** The patient received the PD-1 inhibitor on the first day and then was treated with SBRT (3 doses of 8 Gy, daily) for one metastatic lesion in the right lung. On the second day after SBRT, GM-CSF 200 μg daily was subcutaneously injected for 2 weeks. This course was repeated every 3 weeks and three courses of triple-combination therapy were administrated in total. Then the patient continued to receive two cycles of sintilimab (200 mg/q3w) after triple-combination therapy. **(B)** The patient experienced tumor progression after chemoradiotherapy and targeted therapy. After three cycles of triple-combination therapy and two cycles of sintilimab, the patient's progression-free survival period reached 4 months and eventually died of respiratory failure.

**Figure 2:**
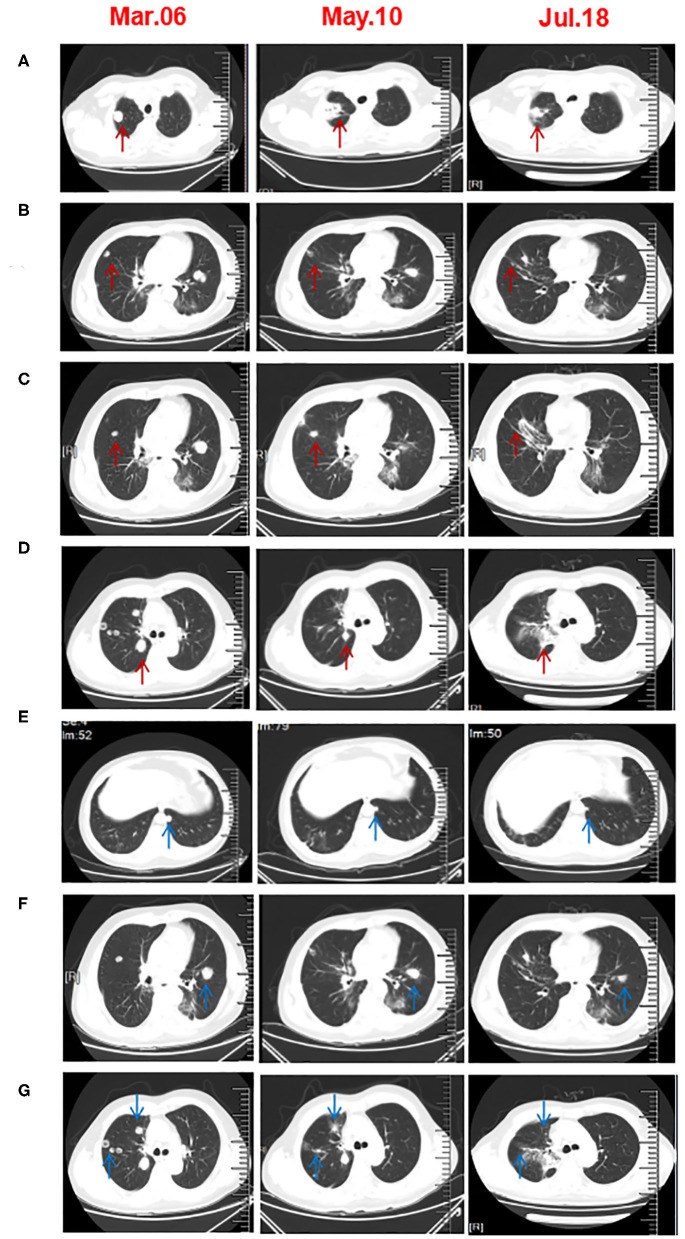
Chest CT scans before and after three cycles of triple-combination therapy and two cycles of sintilimab treatment. **(A–D)** CT revealed that the irradiated right lung lesions shrunk or even disappeared. The arrow in A is the first lesion of the right lung SBRT. The arrow in **(B,C)** are the second lesions of the right lung SBRT. The arrow in **(D)** is the third lesion of the right lung SBRT. **(E,F)** The unirradiated metastatic lesion of the lung was significantly smaller than that before treatment. **(G)** After five cycles of treatment, the CT scan showed that the unirradiated lesions in right lung had disappeared.

**Figure 3:**
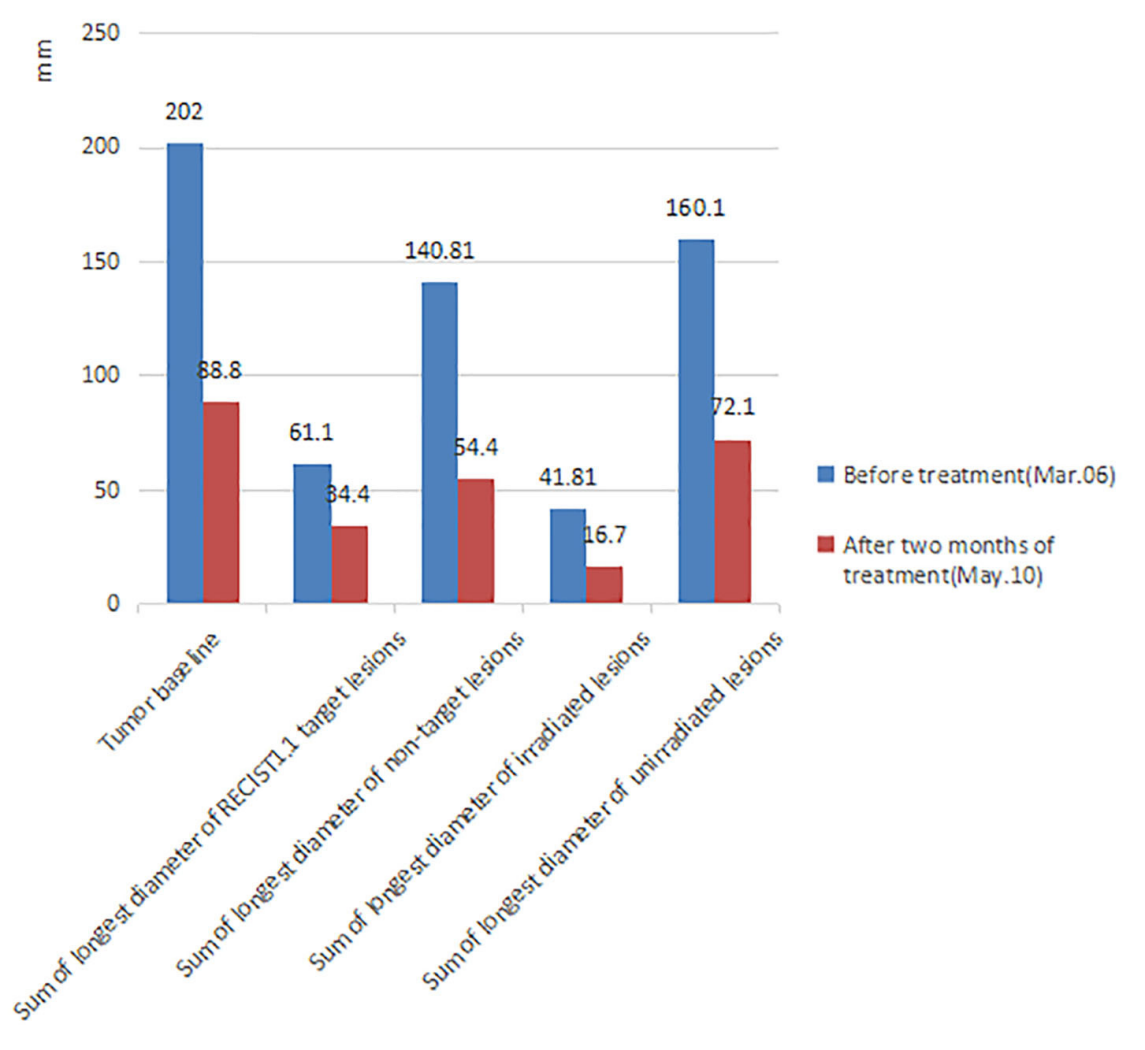
Comparison of tumor burden before treatment and after 2 months of triple-combination therapy. After 2 months of treatment, the patient's tumor burden significantly reduced (The tumor volume data was measured by two doctors, the measurement error of each lesion is <2 mm and we finally took the average value). **Tumor baseline**: Sum of longest diameter of all measurable lesions. **RECIST1.1 Target lesions**: According to the standard of RECIST1.1, each organ can select at most two lesions as target lesions, so we randomly selected two lung metastatic lesions as target lesions before treatment. **Non-target lesions**: All measurable metastatic lesions except target lesions. **Irradiated lesions**: All the SBRT lesions. Considering that the regression of the lesion after radiotherapy will affect the real curative effect, the target lesions is not selected as the SBRT lesions. **Unirradiated lesions**: All measurable metastatic lesions except SBRT lesions.

In 2018, a 57-year-old non-smoker male patient was diagnosed with 90-mm-long ESCC with multiple lymph nodes and lung metastases. Immunohistochemical staining of the tumor tissue showed that the PD-L1 expression was <1% (Supplementary Figure 1). The patient received intensity-modulated radiotherapy (IMRT) from February 28 to April 11, 2018, with doses of 60 Gy/28 f in primary esophageal tumor area and 56 Gy/28 f in metastatic lymph nodes and 50.4 gy/28 f in mediastinal lymphatic drainage with tumor involvement area. At the same time, chemotherapy with six cycles of nedaplatin (35 mg/m^2^ on d1^−2^) and paclitaxel (135 mg/m^2^ on d1) at 3-week intervals was administered. Unfortunately, the patient's lung lesions progressed 2 months after the end of chemotherapy, indicating primary resistance to first-line chemoradiotherapy. Considering the poor condition, the patient was treated with apatinib, but lung metastases progressed in the 3-month evaluation. The patient was then rechallenged with 3 months of anlotinib, but the lung lesions continued to progress. Consequently, the treatment plan was changed, and the PD-1 inhibitor was combined with radiotherapy and GM-CSF from March 2019. The patient received the PD-1 inhibitor (sintilimab 200 mg) on the first day and then was treated with SBRT (3 doses of 8 Gy, daily) for one metastatic lesion in the right lung. On the second day after radiotherapy, GM-CSF 200 μg daily was subcutaneously injected for 2 weeks. This course was repeated every 3 weeks. Three courses of triple-combination therapy were administrated in total and every course was targeted different metastases with SBRT (Figure 1A, Supplementary Figures 3–5). Imaging assessment was performed after three cycles of triple-combination treatment, which revealed remarkable tumor regression at both the irradiated sites and distant unirradiated sites (Figure 2). However, only the mediastinal lymph nodes enlarged (Supplementary Figures 2B,F). Given the significant reduction of tumor burden (Figure 3), the patient continued to receive two cycles of sintilimab (200 mg/q3w) after triple-combination therapy. Later, the enlarged lymph nodes shrunk, indicating that the prior change in the lesion was pseudo-progression (Supplementary Figures 2D,H). In July 2019, the patient was diagnosed with new brain metastases with a Response Evaluation Criteria in Solid Tumors (RECIST1.1) score of PD (progressive disease) and progression-free survival (PFS) of 4 months (Figure 1B).

**Figure 4:**
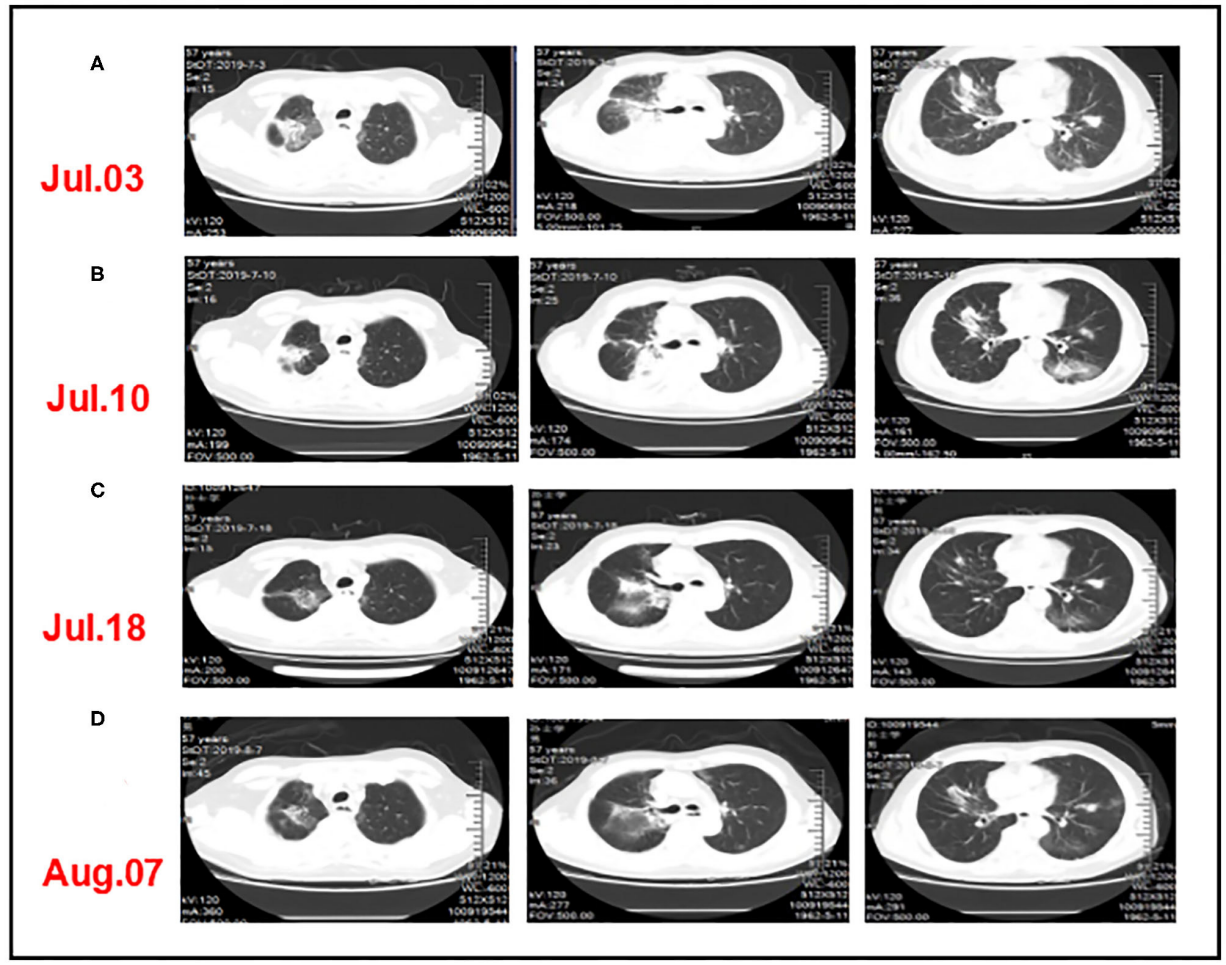
CT comparison of the patient's lung inflammation during anti-infective treatment. **(A)** After three cycles of triple-combination therapy and two cycles of sintilimab monotherapy, chest computed tomography showed inflammatory changes in the lungs and partial lung consolidation. **(B,C)** The chest CT showed that pulmonary infiltration was absorbed after taking a sufficient amount of steroids and antibiotics. **(D)** The patient had a “flare” of pneumonitis symptoms when quickly tapered off steroids.

Grade 1–2 adverse events based on the Common Toxicity Criteria for Adverse Events (version4.0) include fatigue, poor appetite, hypothyroidism, and abnormal liver function. However, these adverse events did not significantly reduce the patient's quality of life. After five cycles of sintilimab, the patient began to have symptoms such as fever, cough, and dyspnea, which gradually aggravated. Chest computed tomography showed inflammatory changes in the lungs and partial lung consolidation (Figure 4A) and sputum culture suggested Acinetobacter epidermidis infection, considering radiation pneumonia combined with bacterial infection, immune-related pneumonia cannot be ruled out. The antitumor therapy was stopped when the patient was diagnosed with grade 3–4 pneumonia. Intravenous methylprednisolone 40 mg every 12 h and antibiotics were administrated. Methylprednisolone was reduced to 40 mg once a day 3 days later. After taking a sufficient amount of steroids and antibiotics for 2 weeks, the patient's symptoms improved significantly and methylprednisolone was reduced to oral 20 mg daily. The chest CT showed that pulmonary infiltration was absorbed and the patient was discharged (Figures 4B,C). However, he had a “flare” of symptoms of pneumonia because he did not follow doctor's advice to slowly tapered off steroids but direct deactivation. We followed up the patient's chest CT (Figure 4D) and continued to administrate steroids and antibiotics. Eventually, the patient and family members refused ventilator-assisted ventilation and died of respiratory failure in August 2019 (Figure 1B).

## Discussion

In recent years, immune checkpoint inhibitors have shown encouraging results in the treatment of metastatic esophageal cancer (16–18). In the KEYNOTE-180 study, a clinically meaningful antitumor activity was observed, the ORR of ESCC patients (14.3%) was higher than that of adenocarcinoma patients (5.2%). The ORR of PD-L1-positive (CPS ≥ 10) population was higher than that of PD-L1-negative (CPS <10) population (14 vs. 6%) (17). The KEYNOTE-181 was a phase 3 trial where pembrolizumab was used in the second line of therapy in patients with advanced or metastatic ESCC or adenocarcinoma/Siewert-type gastroesophageal junction(GEJ) tumors. In the subgroup of PD-L1 CPS ≥ 10, pembrolizumab treatment showed significant benefits in mOS compared with chemotherapy (9.3 and 6.7 months, respectively) (7). In the ATTRACTION-3 study, patients with advanced ESCC refractory or intolerant to previous chemotherapy treatment with nivolumab had achieved a significant improvement in OS and safety profile vs. chemotherapy. It was also showed that the survival benefit of nivolumab was not related to tumor PD-L1 expression, but the patients with PD-L1 expression ≥1% had a 15% lower risk of death than the ones with PD-L1 expression <1% (18).

A phase I study investigated the efficacy and safety of camrelizumab in ≥2 line treatment of ESCC. The treatment with camrelizumab resulted in an ORR of 33.3%, a disease control rate (DCR) of 56.7%, and median PFS of 3.6 months. The incidence of treatment-related adverse events (TRAEs) and grade 3 TRAEs was 83.3 and 10%, respectively. Notably, the disease control rate was 33.3% in PD-L1-negative tumors and 66.7% in PD-L1-positive tumors (19). ESCORT was a phase III trial that evaluated the efficacy and safety of camrelizumab vs. chemotherapy for locally advanced or metastatic ESCC that progressed after first-line treatment, regardless of PD-L1 expression (NCT03099382). The final results of this study were reported at the 15th OESO World Conference. Camrelizumab provided a better survival benefit compared with chemotherapy with PD-L1 ≥ 1% (mOS: 9.2 vs. 6.3 months). The ORR was 20.2% in the study group and 6.4% in the control group. Currently, a phase 3 trial is ongoing to compare cisplatin combined with paclitaxel(TP) plus sintilimab with TP as the first-line treatment in patients with locally advanced unresectable or metastatic ESCC (CTR20181308). Although a large proportion of patients with ESCC have tumors with PD-L1 expression (18.4–82.8%) (20), how PD-L1-negative patients can benefit from immunotherapy needs to be explored.

Strategies to combine other treatment modalities such as radiotherapy are being investigated as means of improving the response rates to a PD-1/PD-L1 inhibitor (21). SBRT can cause more immunogenic death of tumor cells, promote tumor-associated antigen release and presentation, and induce stronger systemic antitumor effects (22). This response can be augmented by the addition of systemic immune-enhancement measures, such as the use of GM-CSF or PD-1/PD-L1 inhibitors (8, 9). Radiotherapy also significantly increases the infiltration of immune cells, thus changing the “immune desert” tumor microenvironment into “immune-inflamed” one (23, 24). Radiotherapy can not only promote antitumor immunity but also produce an immunosuppressive effect. PD-L1 expression can be significantly upregulated by radiotherapy (25), but this negative effect can be offset when combined with anti-PD-1/PD-L1 therapy. In addition, SBRT avoids lymphopenia, indicating a better combination strategy compared with conventionally fractionated radiotherapy or chemotherapy (26).

Several prospective clinical studies showed the safety and efficiency of SBRT combined with a PD-1 inhibitor. A phase I prospective clinical trial of SBRT combined with pembrolizumab in advanced solid tumors showed that the overall ORR was 13.2% with acceptable toxicity (27). Another phase II trial study (PEMBRO-RT) titled “SBRT (3 doses of 8 Gy) sequential pembrolizumab control single drug pembrolizumab in the treatment of advanced non-small cell lung cancer (NSCLC),” the ORR after 12 weeks was 36% in the study group vs. 18% in the control group. The subgroup analysis showed that PD-L1-negative patients benefited the most from radiotherapy without any increase in toxicity (10).

GM-CSF can promote the proliferation of dendritic cells and M1-type macrophages, and enhance antigen presentation to amplify the immune effect of the body (28, 29). The results of a clinical trial of GM-CSF combined with immune checkpoint inhibitors for advanced metastatic melanoma showed that the immune response disease control rate after 24 weeks was 41% and the ORR was 32% (11). The application of a PD-1 inhibitor combined with GM-CSF in the treatment of advanced biliary cancer was found to be safe and effective in a phase II study (14). A prospective study in 2015 showed that local radiotherapy combined with GM-CSF reinforced antitumor effects, inducing tumor regression outside the radiation field, which was called the abscopal effect (13).

In the present case, the lung lesions significantly reduced after three cycles of triple-combination therapy (Figure 2), but the mediastinal lymph nodes enlarged after three cycles of triple-combination therapy (Supplementary Figure 2). The use of PD-1 inhibitor was continued as maintenance treatment, and the lymph nodes shrunk 3 months later (Supplementary Figure 2), which might indicated pseudo-progression in lymph nodes related to T-cell infiltration rather than tumor cell proliferation (30). Unfortunately, we did not perform endoscopic lymph node aspiration for further confirmation. Dynamic efficacy predictors during cancer treatment were important areas of exploration. Some studies confirmed that high levels of tumor-infiltrating lymphocytes were associated with better survival in patients with ESCC (31, 32). Furthermore, several reports indicated that the efficacy of PD-1 inhibitors might be related to peripheral blood lymphocytes. Inflammatory markers such as neutrophil-to-lymphocyte ratio (NLR), platelet-to-lymphocyte ratio (PLR), and lactate dehydrogenase (LDH) may be potential predictive and prognostic factors related to immunotherapy, as shown in recent studies (33–35). The NLR/PLR was defined as an absolute neutrophil/platelet count divided by an absolute lymphocyte count. However, no consistent cutoff values were obtained (34–36). In the present case, the NLR changes did not respond to the treatment effect (Supplementary Figure 7), but the PLR and LDH level decreased during the evaluation after two and three cycles of treatment, indicating the therapeutic effect (Supplementary Figure 8).

Although we innovatively used triple-combination therapy and achieved short-term benefits in this case, we do note that this patient had severe pneumonia which led to his death, suggesting that we should pay more attention to the safety of combination therapy. Some evidence has shown that administration of immune checkpoint inhibitors (ICI) after radiotherapy of lung lesions may cause recall effects (37, 38). Study showed that immune-related (IR) pneumonitis was more common in NSCLC patients treated with ICI who received curative-intent chest radiotherapy, but no radiotherapy parameter was significantly associated with IR pneumonitis (39). The PACIFIC study showed the safety of radiotherapy combined with ICI. Compared with the placebo group, the incidence of pneumonia or radiation pneumonitis in the durvalumab group was 33.9 and 24.8%, and that in grades 3 and 4 was 3.4 and 2.6%, respectively (40). PEMBRO-RT study showed that the incidence of pneumonia in the experimental group was more than that in the control group, but there was no significant difference in the grade 3 to 5 pneumonia (10). The sequence of radiotherapy combined with ICI is still controversial and the use of ICI after radiotherapy may reduce severe pneumonia just as PACIFIC and PEMBRO-RT study did. But KEYNOTE-799 indicated that chemoradiotherapy and simultaneous ICI were well tolerated, and the incidence of pneumonia above grade 3 was 3.4% (41).

In this case, the pneumonia was related to radiation dose of the right lung, for the right lung had been irradiated in 2018 and received three times of SBRT in 2019 (Supplementary Figures 3–6). It is unclear whether the ICI and GM-CSF can aggravate the initial lung injury caused by radiation. In the clinical course, the corticosteroids played important role in the treatment of pneumonia but rebound effects can occur if incorrect use of corticosteroids.

In summary, the triple-combination therapy was effective in the treatment of chemotherapy-refractory and PD-L1-negative metastatic ESCC, the suggested mechanism might involve the radio-sensitization of anti-PD-1 immunotherapy. Safety should be paid more attention to the combination therapy. Therefore, more clinical researches are needed to explore the efficacy and safety of triple-combination therapy and our related clinical research is ongoing (42) (chictr.org.cn No. ChiCTR 1900020175).

## Data Availability Statement

All datasets presented in this study are included in the article/Supplementary Material.

## Ethics Statement

The studies involving human participants were reviewed and approved by Ethics Committee of the Second Affiliated Hospital of Soochow University. The patients/participants provided their written informed consent to participate in this study. Written informed consent was obtained from the individual(s) for the publication of any potentially identifiable images or data included in this article.

## Author Contributions

All authors listed have made a substantial, direct and intellectual contribution to the work, and approved it for publication.

## Conflict of Interest

The authors declare that the research was conducted in the absence of any commercial or financial relationships that could be construed as a potential conflict of interest.
