# Supplementary material for: Anti-PD-1 Immunotherapy Combined With Stereotactic Body Radiation Therapy and GM-CSF as Salvage Therapy in a PD-L1-Negative Patient With Refractory Metastatic Esophageal Squamous Cell Carcinoma: A Case Report and Literature Review
Source: Front Oncol. 2020 Sep 2;10:1625. doi: 10.3389/fonc.2020.01625 (PMC7493754; doi:10.3389/fonc.2020.01625)
Supplement: Supplementary file 1 [file Data_Sheet_1.pdf]

Supplementary Material

Supplementary Figures

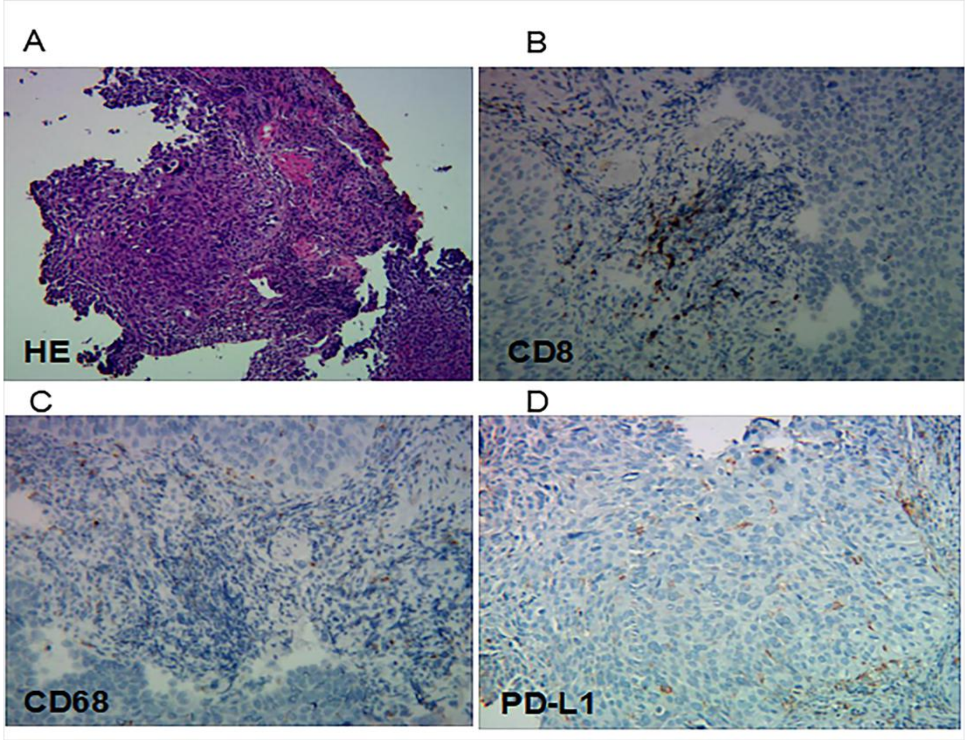

Supplementary Figure 1. Pathology and immunohistochemical staining of the patient's esophagus biopsy specimen. (**A**) Esophageal squamous cell carcinoma (hematoxylin and eosin). (**B**) The CD8 staining was positive. (**C**) The CD68 staining was negative. (**D**) The PD-L1 expression of the patient sample was <1%.

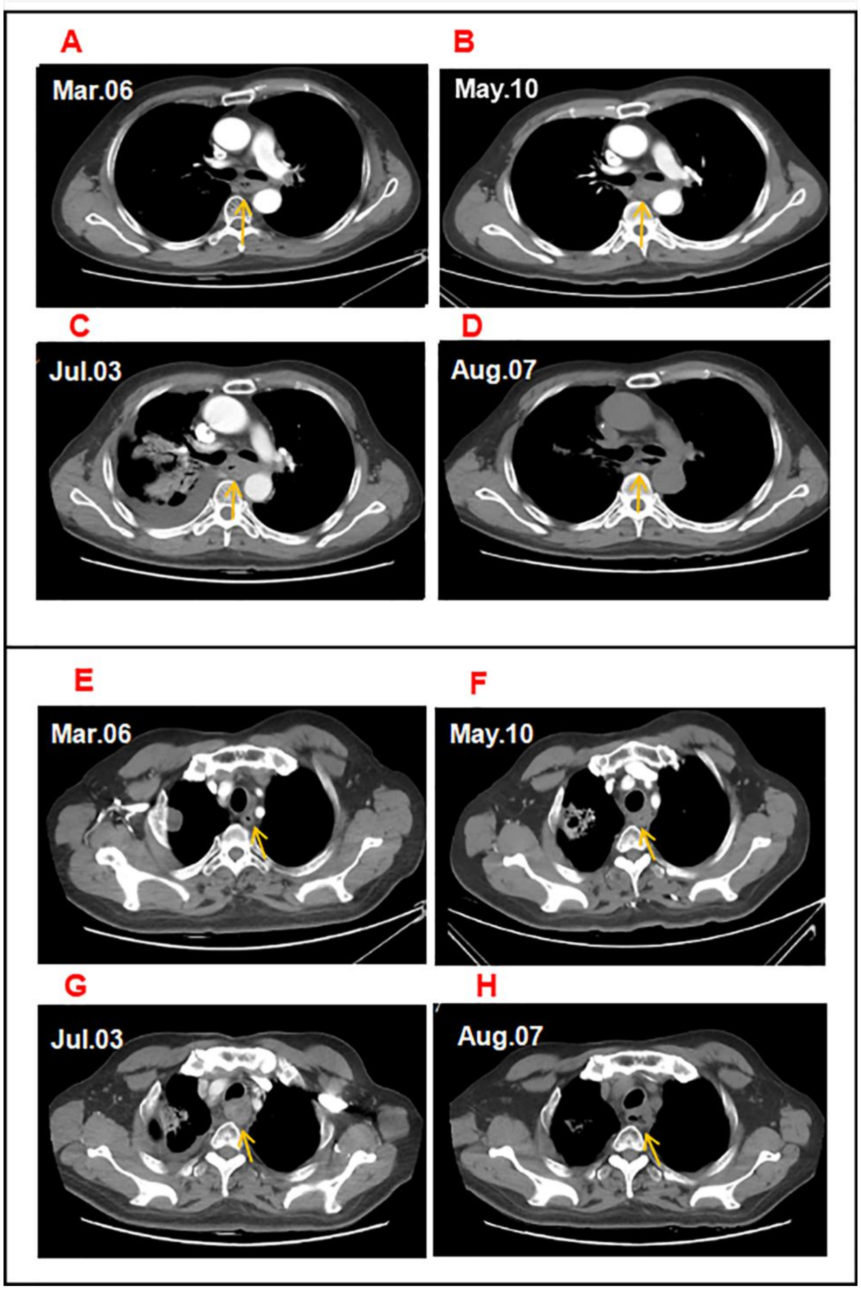

Supplemental Figure 2. Comparison of mediastinal lymph nodes in chest CT during treatment. (**A**、**E**)Before the triple-combination treatment, the CT image showed that the patient had mediastinal enlarged lymph nodes. (**B**、**C**、**F**、**G**)After three cycles of triple-combination therapy and two cycles of sintilimab monotherapy， the mediastinal lymph nodes enlarged. (**D**、**H**) On a follow-up CT scan of the chest performed in August 2019, the enlarged lymph nodes shrunk, indicating that the prior change in the lesion was pseudo-progression.

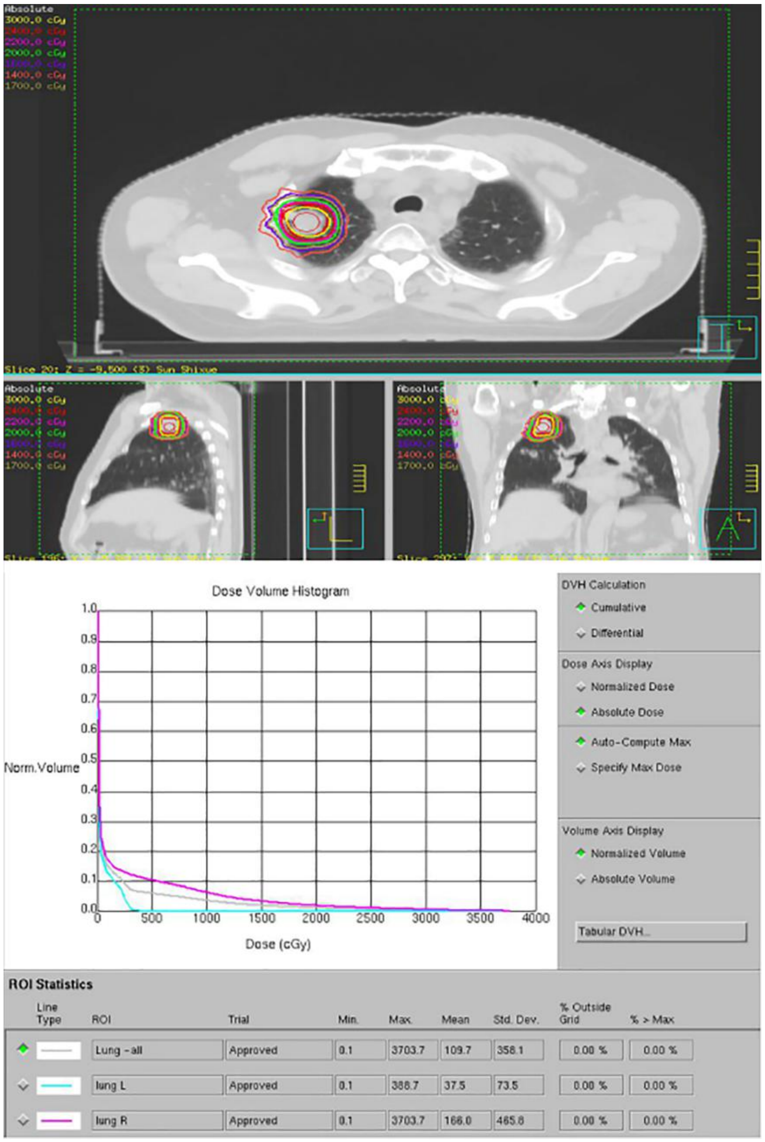

Supplemental Figure 3. SBRT plan of the first irradiated lesion and histogram of lung dose volume.

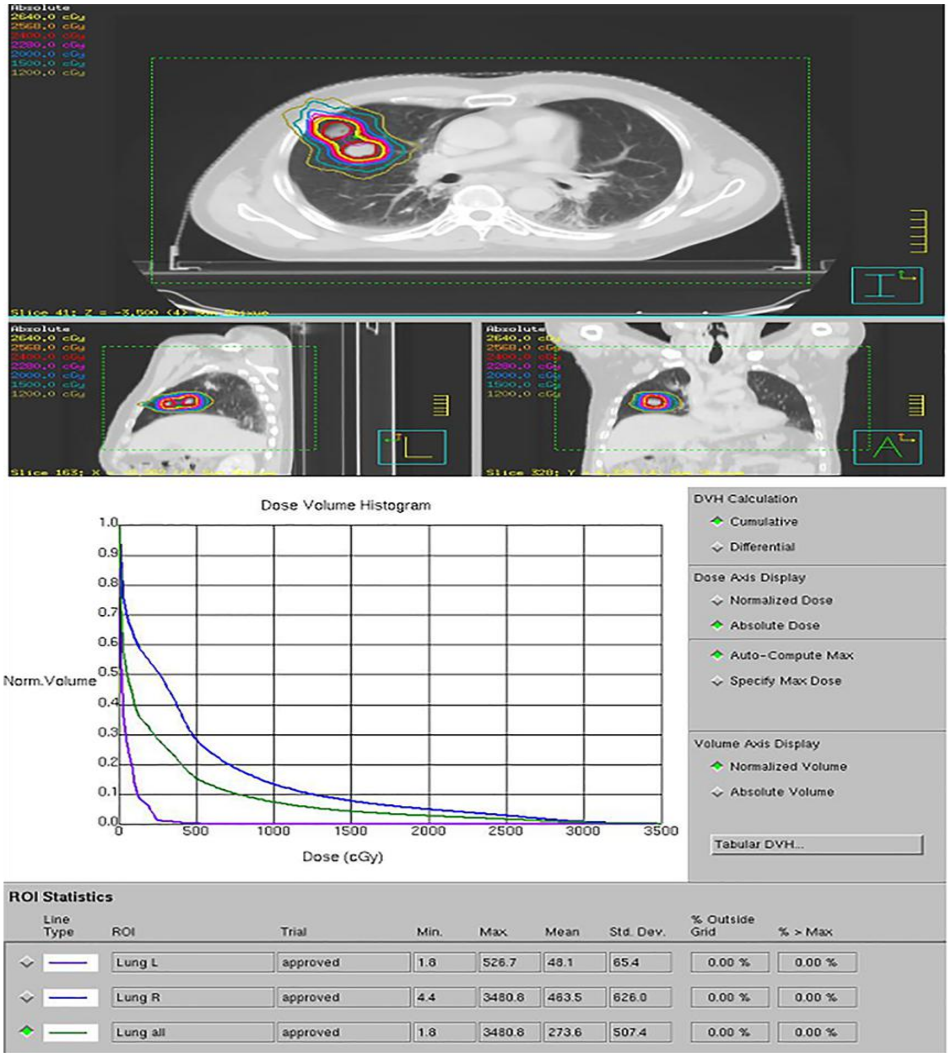

Supplemental Figure 4. SBRT plan of the second irradiated lesion and histogram of lung dose volume.

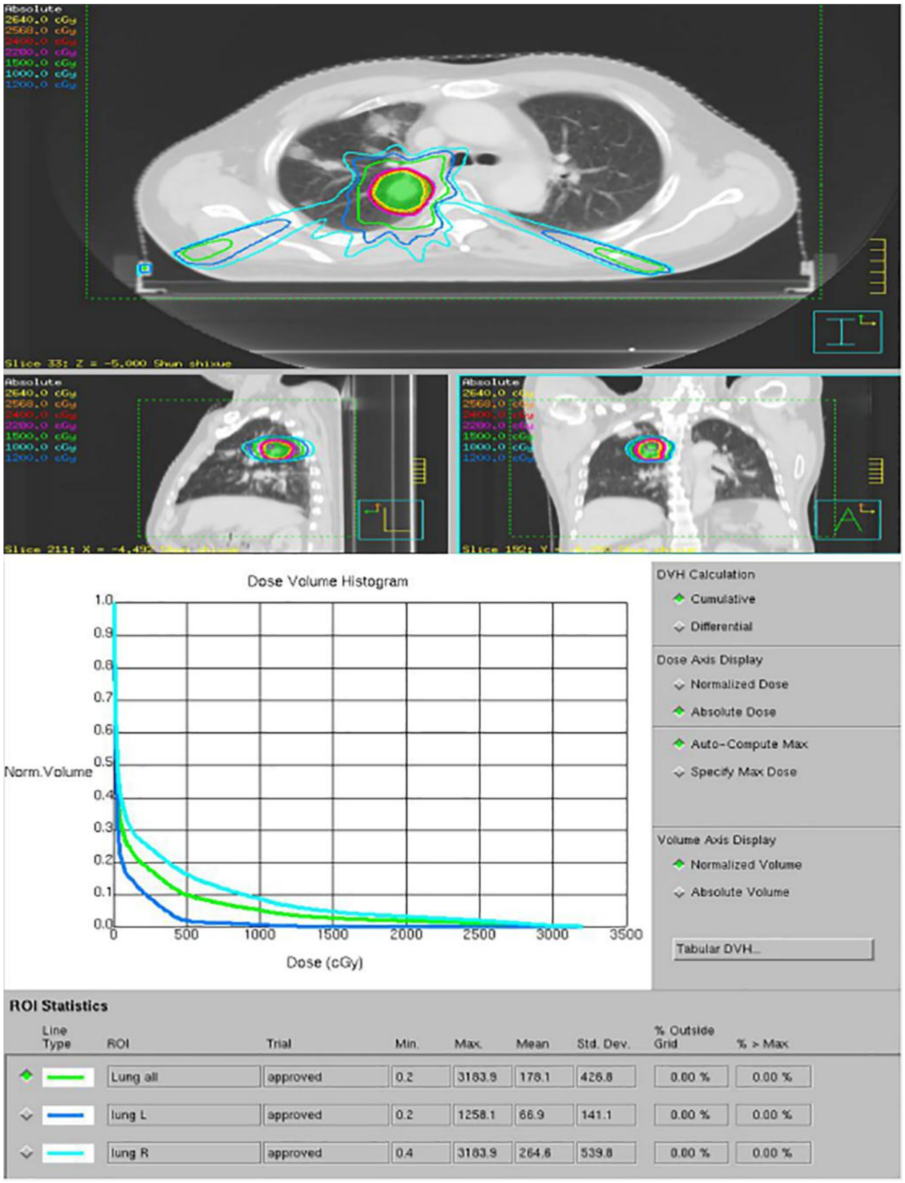

Supplemental Figure 5. SBRT plan of the third irradiated lesion and histogram of lung dose volume. In order to protect the spinal cord, the dose of lung radiation had to be increased.

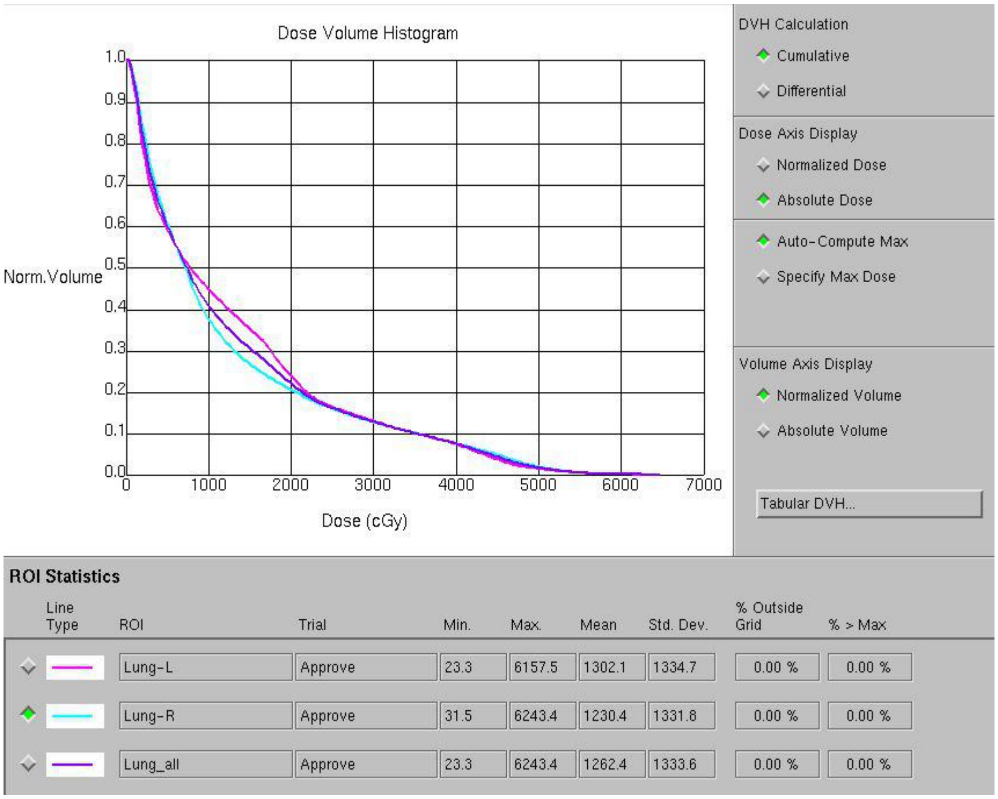

Supplemental Figure 6. Pulmonary dose volume histogram of initial radiotherapy for esophageal cancer in 2018.

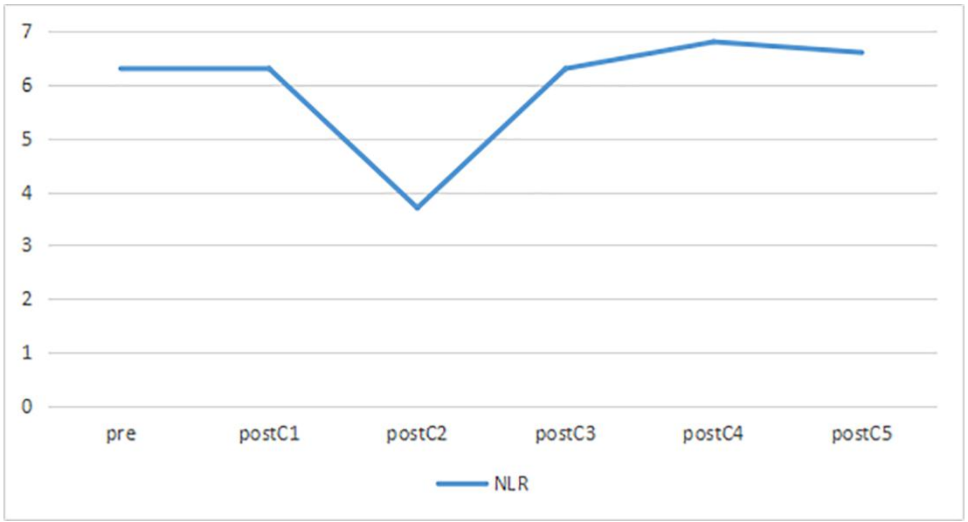

Supplemental Figure 7. The time-series behavior of neutrophil-to-lymphocyte ratio(NLRseries).

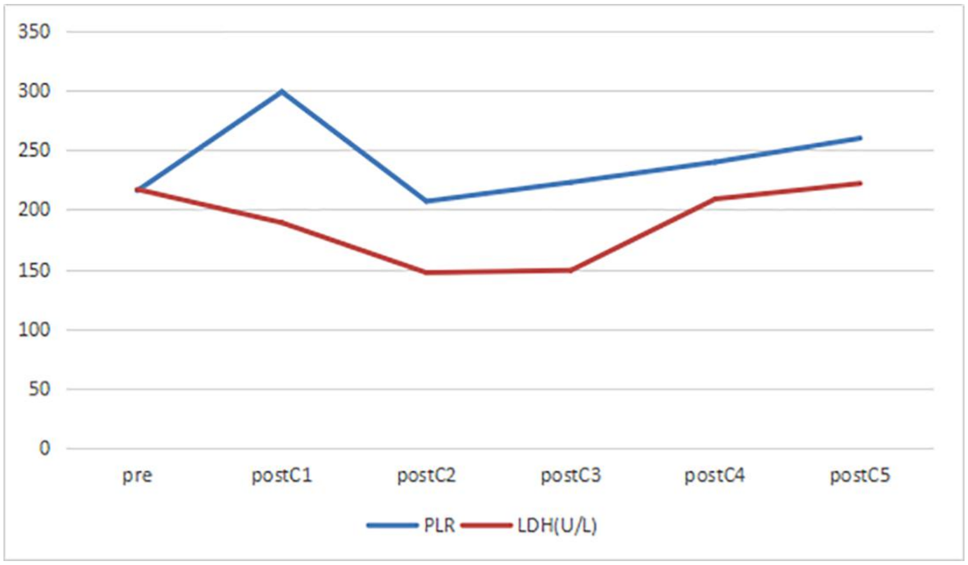

Supplemental Figure 8. The time-series behavior of neutrophil-to-lymphocyte ratio(NLRseries).
